# Stage-specific vaginal microbial alterations in pregnancy-associated vulvovaginal candidiasis

**DOI:** 10.1128/spectrum.02144-25

**Published:** 2025-10-20

**Authors:** Zhe Li, Yiwen Zhang, Xiujie Li, Shuisheng Zhou, Qian Gao, Cailin Wu, Lulu Meng

**Affiliations:** 1 Department of Obstetrics, The Third Affiliated Hospital of Sun Yat-sen University 26469 , Guangzhou, Guangdong, China; 2 High-Risk Perinatal Medicine Center, The Third Affiliated Hospital of Sun Yat-sen University 26469 , Guangzhou, Guangdong, China; 3 Department of Gynecology, The University of Hong Kong-Shenzhen Hospital 444333, Shenzhen, Guangdong, China; 4 Department of Obstetrics, The First Affiliated Hospital of Jinan University 162698, Guangzhou, Guangdong, China; Chengdu University, Chengdu, Sichuan, China

**Keywords:** vaginal microbiota, vulvovaginal candidiasis, pregnancy, co-occurrence network

## Abstract

This study aimed to characterize the vaginal microbiota in women with vulvovaginal candidiasis (VVC) across nonpregnant states and different stages of pregnancy, to explore gestation-specific patterns of microbial dysbiosis. Vaginal samples were collected from nonpregnant and pregnant women with clinically diagnosed VVC, including cases in early, mid-, and late pregnancy. Microbial composition was assessed using 16S rRNA gene sequencing targeting the V3–V4 region. Alpha and beta diversity metrics were calculated to evaluate microbial richness, evenness, and structural differences. Linear discriminant analysis effect size (LEfSe) analysis and microbial co-occurrence network modeling were performed to identify differentially abundant taxa and examine community connectivity. VVC-associated microbial communities exhibited distinct diversity and structural features across gestational stages. Notably, increased Shannon and Simpson indices were observed in early and mid-pregnancy VVC cases. Beta diversity analysis revealed significant differences between VVC and healthy controls within each trimester. LEfSe analysis consistently identified a decrease in *Lactobacillus* in VVC patients. Microbial co-occurrence networks in VVC groups showed higher modularity in early pregnancy, whereas networks in mid- and late pregnancy exhibited reduced connectivity and lower average degree compared with healthy controls. *Lactobacillus* showed reduced network connectivity in early and late pregnancy, but its integration was markedly increased in mid-pregnancy VVC cases. This study demonstrates that vaginal microbial dysbiosis in VVC exhibits distinct, gestational stage-specific features. While *Lactobacillus* reduction is a consistent hallmark, microbial diversity and network structure vary notably across trimesters, with *Lactobacillus* connectivity also showing significant changes. These findings highlight the importance of gestational age-specific perspectives in both diagnosis and clinical management of VVC.

**IMPORTANCE:**

Vulvovaginal candidiasis (VVC) is highly prevalent in reproductive-age women and poses unique challenges during pregnancy. While previous studies have explored the vaginal microbiota in healthy pregnancies or in VVC-affected nonpregnant women, few have comprehensively evaluated how VVC-associated dysbiosis evolves across gestational stages. In this study, we revealed that microbial diversity, taxonomic composition, and ecological network structures differ significantly across trimesters in women with VVC. Notably, we found that the topological role of *Lactobacillus*—central to vaginal health—changes dynamically during pregnancy, indicating gestational stage-specific vulnerabilities in microbial resilience. These findings offer new insight into how pregnancy shapes the vaginal ecosystem under pathological conditions and highlight the need for trimester-tailored strategies in the management and prevention of VVC to better protect maternal and neonatal outcomes.

## INTRODUCTION

Vulvovaginal candidiasis (VVC) is the second most prevalent vaginal infection following bacterial vaginosis (1). It is characterized by vaginal microbiota disorder and concurrent overgrowth of *Candida* spp., including *Candida albicans*, *Candida glabrata*, and *Candida tropicalis* (2). Antimicrobial use, diabetes, estrogen levels, immunosuppressive treatments, glucocorticoids, psychosocial stress, and sexual activity have been identified as risk factors for VVC by contributing to vaginal microbial imbalance (3–5).

In healthy women, the vaginal microbiota is typically dominated by *Lactobacillus* species, which help maintain an acidic environment and inhibit the growth of opportunistic pathogens (6, 7). Based on dominant taxa, the vaginal microbiome is classified into five community state types (CSTs). CSTs I–III and V are each dominated by a single *Lactobacillus* species (*Lactobacillus crispatus*, *Lactobacillus gasseri*, *Lactobacillus iners*, or *Lactobacillus jensenii*), whereas CST IV exhibits low *Lactobacillus* abundance and higher diversity of anaerobic bacteria, reflecting dysbiosis (8–10).

Pregnancy further modifies the vaginal microbiota (11, 12). Elevated estrogen promotes vaginal glycogen accumulation, which is metabolized by *Lactobacillus* to produce lactic acid, reinforcing a protective acidic environment. However, glycogen can also serve as a nutrient source for *Candida*, facilitating fungal overgrowth (13). The incidence of VVC during pregnancy has been reported to be approximately 20% (14), driven by hormonal fluctuations, immune modulation, and gestational diabetes (15).

Importantly, maternal VVC has been associated with adverse pregnancy outcomes such as preterm labor, premature rupture of membranes, chorioamnionitis, and low birth weight (16–20). In late pregnancy, it may also be linked to vertical transmission of *Candida*, potentially predisposing neonates to early-onset mucocutaneous infections such as oral thrush and diaper dermatitis (21). Despite these risks, few studies have systematically compared the vaginal microbiota of healthy and VVC-affected women across different trimesters or relative to nonpregnant women.

Current management of VVC in pregnancy primarily relies on topical azole antifungals, such as clotrimazole, which are considered safe for use during gestation (22, 23). Probiotics have also been investigated as an adjunct therapy to restore *Lactobacillus*-dominated communities, but findings remain inconsistent, with no consensus on optimal strains, dosage, or timing (21). Characterizing the vaginal microbiota during pregnancy is therefore essential to provide foundational data that may inform the design of potential future interventions.

In this context, this study aimed to characterize the vaginal microbiota in women with and without VVC across different gestational stages, as well as in nonpregnant women with VVC, while also examining the longitudinal dynamics of microbial communities in healthy and affected pregnancies. This approach was designed to elucidate how VVC-associated dysbiosis evolves with gestational age and physiological status, providing a microbial basis to inform future gestation-specific prevention and management strategies.

## MATERIALS AND METHODS

### Study design and participants

A total of 149 women were enrolled in this study from The Third Affiliated Hospital of Sun Yat-sen University and The University of Hong Kong–Shenzhen Hospital, and their phenotypic information, including height, weight, age, disease history, etc., is summarized in Table S1. This cohort included 27 nonpregnant women with VVC (NP-VVC) and 122 pregnant women. Among the pregnant participants, 62 were diagnosed with VVC at different gestational stages: 20 in early pregnancy (EP-VVC, ≤13^+6^ weeks), 19 in mid-pregnancy (MP-VVC, 14–27^+6^ weeks), and 23 in late pregnancy (LP-VVC, ≥28 weeks). The remaining 60 pregnant women were healthy controls, with 20 participants in each trimester: early pregnancy (EP-C), mid-pregnancy (MP-C), and late pregnancy (LP-C).

### Inclusion and exclusion criteria

The inclusion criteria for pregnant women were as follows: (i) Chinese women who were pregnant with a single fetus and (ii) natural pregnancy. The exclusion criteria for pregnant participants were as follows: (i) use of antibiotics, antifungal agents, or probiotics during pregnancy; (ii) hypertension, diabetes mellitus, hyperthyroidism, hypothyroidism, autoimmune disease, or other endocrine and metabolic disorders; (iii) gestational hypertensive disease, gestational diabetes mellitus, or other pregnancy-related complications; (iv) history of transfusion, organ transplantation, or immunotherapy; and (5) recurrent VVC or co-infections, such as bacterial vaginosis, trichomoniasis, or other clinically diagnosed vaginal infections. The exclusion criteria for nonpregnant participants were as follows: (i) use of antibiotics, antifungal agents, or probiotics within 14 days prior to enrollment; (ii) hypertension, diabetes mellitus, hyperthyroidism, hypothyroidism, autoimmune disease, or other endocrine and metabolic disorders; (iii) history of transfusion, organ transplantation, or immunotherapy; and (iv) recurrent VVC or co-infections, such as bacterial vaginosis, trichomoniasis, or other clinically diagnosed vaginal infections.

### VVC diagnostic criteria

VVC was diagnosed based on both clinical presentation and microscopic examination of vaginal secretions. Clinical symptoms included external dysuria, vulvar pruritus, pain, swelling, and redness. Indicative signs comprised vulvar edema, fissures, excoriations, and thick curdy vaginal discharge (24). Microscopic examination using the wet mount method was performed to detect budding yeasts, hyphae, or pseudohyphae. A diagnosis of VVC was confirmed when characteristic clinical symptoms and signs were observed in conjunction with the presence of yeasts, hyphae, or pseudohyphae.

### Vaginal secretion collection

Both nonpregnant and pregnant women were instructed to abstain from sexual activity, refrain from cleansing the vulva or vagina, and avoid using vaginal medication within 48 hours prior to sample collection. To minimize potential variability due to diurnal fluctuations, all samples were collected in the morning. For nonpregnant women, sampling was performed during the follicular phase to control for hormonal influences on the vaginal microbiota. A vaginal speculum was used to expose the vagina, and aseptic cotton swabs were used to collect vaginal discharge from one-third of the lateral vaginal wall. One of the swabs, soaked in 0.9% normal saline solution, was utilized for microscopic identification of pathogenic microorganisms. The remaining specimens were promptly preserved at −80°C until subsequent 16S rRNA gene sequencing analysis.

### Experimental procedure

#### 16S rRNA sequencing

For vaginal swabs, DNA was extracted using the cetyltrimethylammonium bromide method. The V3–V4 region of the bacterial 16S rRNA gene was amplified using primers 341F (CCTAYGGGRBGCASCAG) and 806R (GGACTACNNGGGTATCTAAT). Sequencing libraries were constructed and sequenced on an Illumina platform. Quality control steps, including paired-end read merging with FLASH, quality filtering with fastp, and chimera removal using VSEARCH, were performed. Amplicon sequence variants (ASVs) were generated using DADA2, and taxonomic annotation was conducted against the SILVA database using QIIME2.

#### Alpha diversity

Alpha diversity reflects the complexity of microbial communities within a sample. Community richness refers to the number of distinct taxa present, while community diversity considers both richness and the relative abundance distribution of these taxa. In this study, four indices were calculated using QIIME2: Chao1 and Observed_species for richness, and Shannon and Simpson for diversity (Chao1: https://scikit.bio/docs/latest/generated/skbio.diversity.alpha.chao1.html; Observed_species: https://scikit.bio/docs/latest/generated/skbio.diversity.alpha.observed_species.html; Shannon: https://scikit.bio/docs/latest/generated/skbio.diversity.alpha.shannon.html; Simpson: https://scikit.bio/docs/latest/generated/skbio.diversity.alpha.simpson.html).

#### Beta diversity

To evaluate beta diversity, Principal Coordinates Analysis (PCoA) was conducted based on Bray–Curtis, weighted UniFrac, and unweighted UniFrac distance matrices. Statistical significance of group separation was assessed using Analysis of Similarities (ANOSIM), with corresponding *P* values indicating the extent of compositional dissimilarity across groups.

### Group characteristics analysis

Linear Discriminant Analysis Effect Size (LEfSe) was employed to identify potential microbial biomarkers and characterize metagenomic features (25). The analysis was carried out with the original Python-based LEfSe tool (version 1.1.01) on the MagicPlus platform provided by Novogene (https://magic-plus.novogene.com). Default settings were applied, with the alpha value for the Kruskal–Wallis test set at 0.05 and the logarithmic LDA score threshold at 4.0. Discriminative features were displayed using bar plots and cladograms.

### Co-occurrence network analysis

Spearman’s rank correlation coefficient was calculated among genera with the R package Hmisc based on relative abundance profiles. Genera present in at least 10% of the samples within each group were retained for network construction. Only correlations with |r| ≥ 0.4 and adjusted *P* < 0.05 (Benjamini–Hochberg correction) were considered significant. The global network was visualized with Gephi (v0.3) (26). To compare network topology across groups, subnetwork-level indices were calculated for each sample using the igraph R package (27).

### Vaginal microbiota–perinatal outcome correlations

The top 50 most abundant vaginal genera in each group were correlated with perinatal outcomes using Spearman’s rank correlation. Only correlations with |r| ≥ 0.4 and adjusted *P* < 0.05 (Benjamini–Hochberg correction) were considered significant.

### Statistical analysis

All continuous variables were tested for normality. Normally distributed data are presented as mean ± standard error, and non-normally distributed data as median (interquartile range). Depending on the distribution, comparisons between two groups were performed using independent-sample t-tests or Wilcoxon rank-sum tests, while comparisons among three or more groups were conducted using one-way ANOVA or Kruskal–Wallis tests. Categorical variables were analyzed using the chi-square test. A *P* < 0.05 was considered statistically significant. All data were analyzed using SPSS 29.0.2.0 statistical software (SPSS, Inc., Chicago, IL, USA)

## RESULTS

### Clinical characteristics of each group

The clinical characteristics are shown in Table 1. No significant differences were observed in maternal age, height, BMI, gravidity, parity, or education level among NP-VVC, EP-VVC, and EP-C; MP-VVC and MP-C; or LP-VVC and LP-C (*P* > 0.05). Similarly, maternal clinical outcomes—including gestational age at delivery, mode of delivery (cesarean or vaginal), and incidence of premature rupture of membranes (PROM)—did not differ significantly between EP-VVC and EP-C, MP-VVC and MP-C, or LP-VVC and LP-C (*P* > 0.05). Neonatal clinical outcomes, including birth weight, neonatal intensive care unit (NICU) admission, amniotic fluid clarity, and 1- and 5-minute Apgar scores, did not differ significantly between VVC patients and their corresponding control groups (*P* > 0.05). Within each cohort, there were no significant changes in maternal and neonatal outcomes across gestational stages, either among healthy pregnant women (EP-C, MP-C, LP-C) or among VVC-affected pregnant women (EP-VVC, MP-VVC, and LP-VVC) (*P* > 0.05).

**TABLE 1:** Demographic and clinical characteristics of participants in different groups^*a*^

Category	NP-VVC (*n* = 27)	EP-VVC (*n* = 20)	EP-C (*n* = 20)	MP-VVC (*n* = 19)	MP-C (*n* = 20)	LP-VVC (*n* = 23)	LP-C (*n* = 20)
Maternal demographics							
Age (years)	31.81 ± 5.85	29.20 ± 2.88	30.85 ± 3.57	29.37 ± 4.44	29.85 ± 3.13	31.00 ± 4.80	30.80 ± 3.58
Weight (kg)	54.64 ± 12.02	53.04 ± 7.37	55.10 ± 7.39	58.13 ± 8.54	58.15 ± 6.11	65.13 ± 8.42	66.25 ± 6.81
Height (cm)	158.59 ± 4.00	159.00 ± 5.99	158.30 ± 2.94	158.21 ± 4.44	157.30 ± 6.27	159.22 ± 3.36	157.90 ± 3.95
Gravida	1 (1–1)	1 (1–2)	1 (1–2)	1 (1–1.5)	1 (1–3)	2 (1–2)	2 (1–3)
Para	0 (0–1)	0 (0–1)	0 (0–1)	0 (0–1)	0 (0–1)	0 (0–1)	1 (0–1)
BMI (kg/m^2^)	21.74 ± 4.80	20.95 ± 2.58	21.95 ± 2.63	23.23 ± 3.26	23.50 ± 2.15	25.67 ± 3.03	26.54 ± 2.21
Education—Undergrad	20 (74.07%)	17 (85.00%)	18 (90.00%)	16 (84.21%)	16 (80.00%)	17 (73.91%)	14 (70.00%)
Education—High school	5 (18.52%)	1 (5.00%)	0	3 (15.79%)	3 (15.00%)	4 (17.39%)	3 (15.00%)
Education—Graduate	2 (7.41%)	2 (10.00%)	2 (10.00)	0	1 (5.00%)	2 (8.70%)	3 (15.00%)
Maternal clinical outcomes							
GA at delivery (days)	NA	273.00 (270.09–276.11)	272.65 ± 8.24	273.11 ± 3.81	274.35 ± 6.85	276.78 ± 6.35	275.5 (269.06–278.44)
Delivery mode—cesarean	NA	5 (25.00%)	5 (25.00%)	5 (26.32%)	9 (45.00%)	13 (56.52%)	7 (35.00%)
Delivery mode—vaginal	NA	15 (75.00%)	15 (75.00%)	14 (73.68%)	11 (55.00%)	10 (43.48%)	13 (65.00%)
PROM—no	NA	12 (60.00%)	15 (75.00%)	14 (73.68%)	16 (80.00%)	19 (82.61%)	18 (90.00%)
PROM—yes	NA	8 (40.00%)	5 (25.00%)	5 (26.32%)	4 (20.00%)	4 (17.39%)	2 (10.00%)
Neonatal clinical outcomes							
Fetal weight (kg)	NA	2.94 ± 0.335	2.95 ± 0.39	3.21 ± 0.31	3.45 (3.15–3.56)	3.26 ± 0.43	3.20 ± 0.33
NICU admission—yes	NA	15 (75.00%)	17 (85.00%)	18 (94.74%)	16 (80.00%)	20 (86.96%)	17 (85.00%)
NICU admission—no	NA	5 (25.00%)	3 (15.00%)	1 (5.26%)	4 (20.00%)	3 (13.04%)	3 (15.00%)
Amniotic fluid—clear	NA	17 (85.00%)	18 (90.00%)	18 (94.74%)	19 (95.00%)	21 (91.30%)	16 (80.00%)
Amniotic fluid—turbid	NA	3 (15.00%)	2 (10.00%)	1 (5.26%)	1 (5.00%)	2 (8.70%)	4 (20.00%)
1 min Apgar—6	NA	2 (10.00%)	1 (5.00%)	0	0	0	0
1 min Apgar—8	NA	1 (5.00%)	1 (5.00%)	0	0	0	0
1 min Apgar—10	NA	17 (85.00%)	18 (90.00%)	19	20	23	20
5 min Apgar—10	NA	20	20	19	20	23	20

^*a*^ Gravida: a number to indicate the number of pregnancies a woman has had. Para: the number of liveborn children a woman has delivered. GA, gestational age (days); PROM, premature rupture of membranes; NICU, neonatal intensive care unit; NA, not applicable.

### Characterizing the composition of the vaginal microbiota in different groups

**Fig 1:**
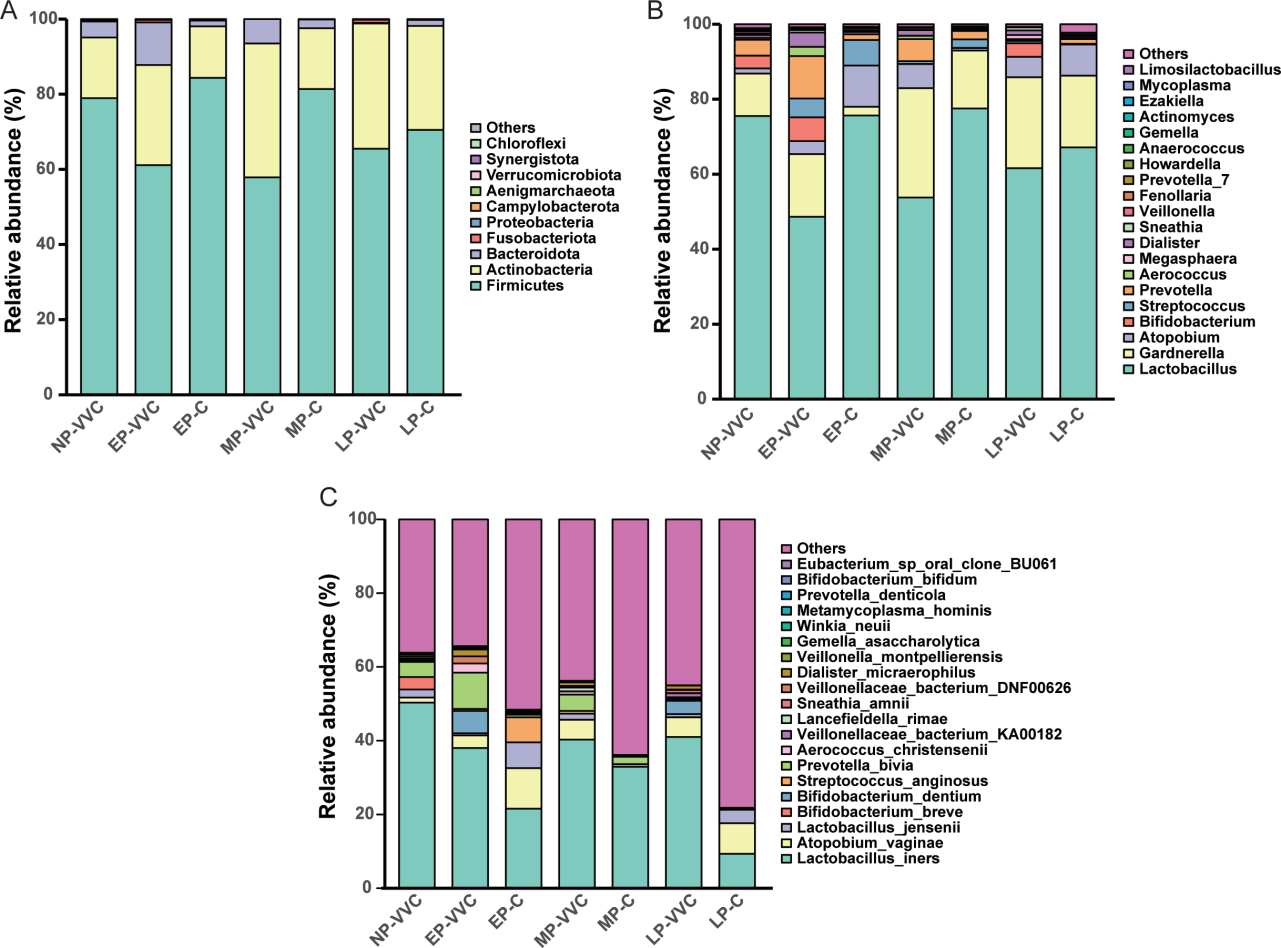
Vaginal microbiota composition across groups at different taxonomic levels. (**A**) Bar chart showing relative abundances of vaginal microbiota at the phylum level in each group. (**B**) Bar chart showing relative abundances of vaginal microbiota at the genus level in each group. (**C**) Bar chart showing relative abundances of vaginal microbiota at the species level in each group.

At the phylum level, the dominant taxa varied by group. In NP-VVC, EP-VVC, and MP-VVC, the three most abundant phyla were *Firmicutes* (78.95%, 61.10%, and 57.87%), *Actinobacteriota* (16.12%, 26.69%, and 35.63%), and *Bacteroidota* (4.27%, 11.34%, and 6.47%), respectively. The corresponding control groups (EP-C, MP-C, and LP-C) were likewise dominated by *Firmicutes* (84.35%, 81.37%, and 70.47%), followed by *Actinobacteriota* (13.73%, 16.21%, and 27.70%) and *Bacteroidota* (1.54%, 2.29%, and 1.61%). In the LP-VVC group, *Firmicutes* remained dominant (65.48%), followed by *Actinobacteria* (33.35%) and *Fusobacteriota* (0.80%) (Fig. 1A; Table S2).

At the genus level, *Lactobacillus* was consistently the most dominant taxon across all groups, with relative abundances ranging from 48.65% to 77.50%. Specifically, its proportions were 75.51% in NP-VVC, 48.65% in EP-VVC, 53.78% in MP-VVC, 61.62% in LP-VVC, 75.66% in EP-C, 77.50% in MP-C, and 67.14% in LP-C. *Gardnerella* ranked second in most groups, with relative abundances of 11.34% in NP-VVC, 16.70% in EP-VVC, 29.17% in MP-VVC, 24.25% in LP-VVC, 2.31% in EP-C, 15.49% in MP-C, and 19.14% in LP-C. *Atopobium* appeared in the top five genera in NP-VVC (1.37%), MP-VVC (6.41%), LP-VVC (5.42%), EP-C (11.02%), MP-C (0.67%), and LP-C (8.29%). *Prevotella* ranked among the top five in NP-VVC (4.22%), EP-VVC (11.29%), MP-VVC (5.92%), EP-C (1.52%), MP-C (2.26%), and LP-C (1.27%). *Bifidobacterium* appeared in the top five in NP-VVC (3.39%), EP-VVC (6.34%), and LP-VVC (3.63%), while *Streptococcus* was among the top five in EP-VVC (5.04%), EP-C (6.79%), and MP-C (2.28%). Other genera, including *Dialister* (MP-VVC 1.47%, LP-VVC 1.23%) and *Ezakiella* (LP-C 0.51%), were occasionally ranked within the top five depending on the group (Fig. 1B; Table S3).

At the species level, *Lactobacillus_iners* was consistently the most dominant taxon across all groups, with relative abundances of 50.34% in NP-VVC, 38.01% in EP-VVC, 40.32% in MP-VVC, 41.01% in LP-VVC, 21.56% in EP-C, 32.93% in MP-C, and 9.33% in LP-C. *Atopobium_vaginae* ranked second in most groups, accounting for 1.36% in NP-VVC, 3.44% in EP-VVC, 5.36% in MP-VVC, 5.34% in LP-VVC, 11.01% in EP-C, 0.66% in MP-C, and 8.28% in LP-C. Other species among the top five varied by group: *Prevotella_bivia* was enriched in NP-VVC (4.10%), EP-VVC (9.85%), MP-VVC (4.42%), EP-C (0.72%), MP-C (1.98%), and LP-C (0.33%), while *Lactobacillus_jensenii* appeared in NP-VVC (2.17%), MP-VVC (1.65%), EP-C (6.97%), and LP-C (3.64%). Several taxa occasionally ranked within the top five in specific groups, including *Bifidobacterium_dentium* (EP-VVC 6.05%; LP-VVC 3.62%), *Aerococcus_christensenii* (EP-VVC 2.48%; MP-C 0.32%), *Dialister_micraerophilus* (LP-VVC 1.08%; MP-C 0.20%), *Bifidobacterium_breve* (NP-VVC 3.39%), *Streptococcus_anginosus* (EP-C 6.78%), *Veillonellaceae_bacterium_KA00182* (LP-VVC 1.07%), *Lancefieldella_rimae* (MP-VVC 1.04%), and *Veillonellaceae_bacterium_DNF00626* (LP-C 0.09%) (Fig. 1C; Table S4).

CSTs were further analyzed, revealing that CST III predominated across all groups. No significant differences were observed among NP-VVC, EP-VVC, and EP-C in the early pregnancy stage, between MP-VVC and MP-C in mid-pregnancy, or between LP-VVC and LP-C in late pregnancy, nor within cohorts across stages (all *P* > 0.05) (Table 2).

**TABLE 2:** Distribution of CSTs across groups^*a*^

Group	CST III n (%)	CST IV n (%)	CST V n (%)
NP-VVC	19 (70.37)	6 (22.22)	2 (7.41)
EP-C	12 (60.00)	7 (35.00)	1 (5.00)
EP-VVC	11 (55.00)	9 (45.00)	0
MP-C	12 (60.00)	8 (40.00)	0
MP-VVC	13 (68.42)	6 (31.58)	0
LP-C	10 (50.00)	8 (40.00)	2 (10.00)
LP-VVC	16 (69.57)	7 (30.43)	0

^*a*^ CSTs were classified based on vaginal microbial composition, and differences across groups were assessed using Fisher’s exact test.

### Alpha-diversity differences

**Fig 2:**
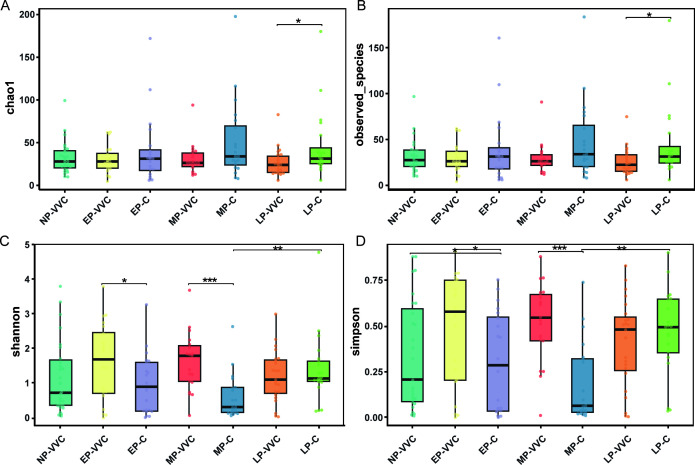
Alpha diversity of vaginal microbiota across groups. (**A**) Chao1 index. (**B**) Observed species index. (**C**) Shannon index. (**D**) Simpson index. Comparisons between two groups were performed using t-tests or Wilcoxon rank-sum tests, and comparisons among three groups were conducted using one-way ANOVA or Kruskal–Wallis tests. *P < 0.05, **P < 0.01, ***P < 0.001.

Chao1, Observed_species, Shannon, and Simpson indices were used to assess alpha diversity of the vaginal microbiota. The LP-C group exhibited higher species richness, as indicated by elevated Chao1 and Observed species indices, compared to the LP-VVC group (*P* < 0.05). In terms of community diversity, the EP-VVC and MP-VVC groups showed higher Shannon and Simpson indices than the EP-C and MP-C groups, respectively (*P* < 0.05) (Fig. 2). Across gestational stages, the MP-C group displayed lower Shannon and Simpson indices than the LP-C group (*P* < 0.05). No significant differences in alpha diversity were observed among the EP-VVC, MP-VVC, and LP-VVC groups (*P* > 0.05).

### Beta-diversity differences

**Fig 3:**
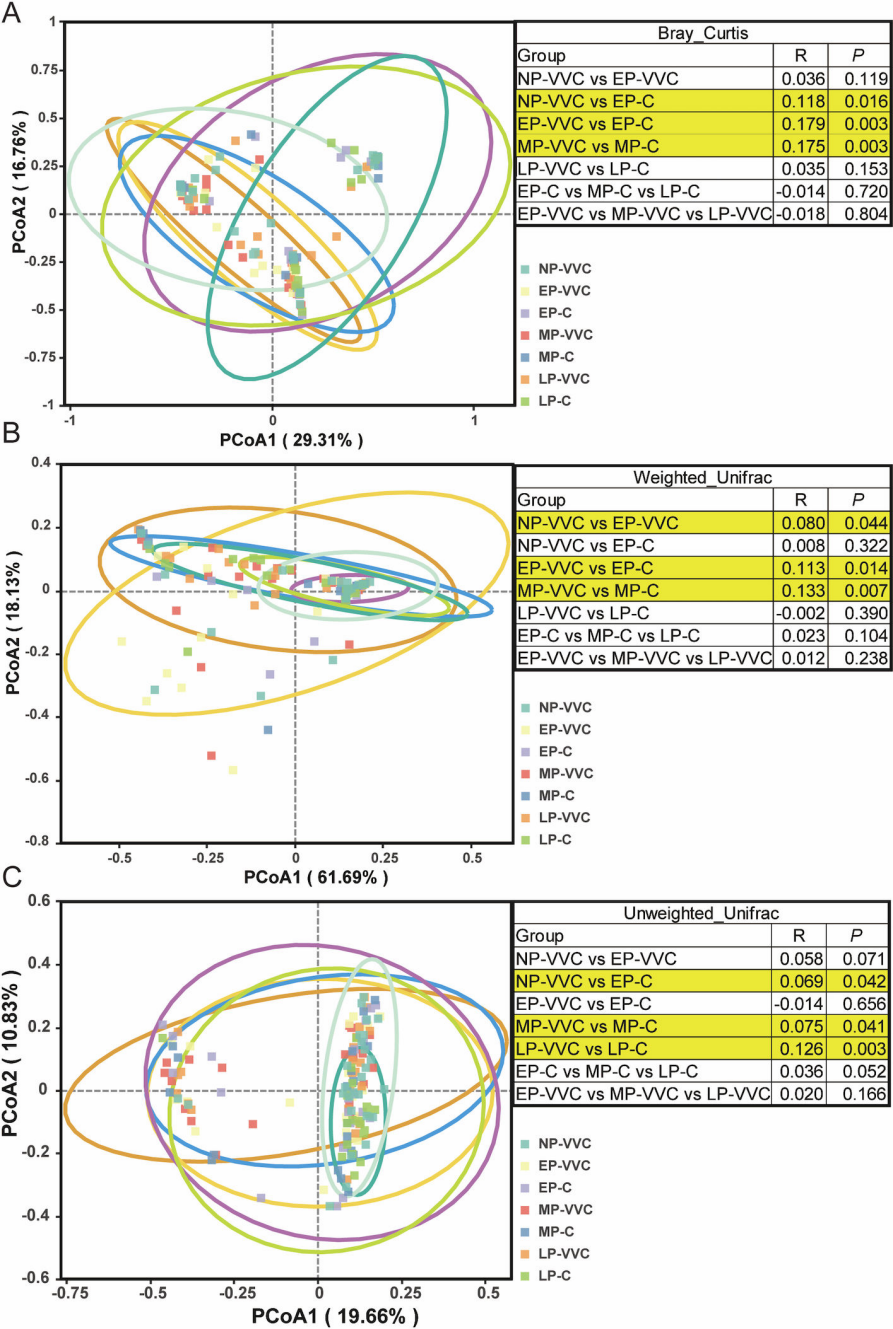
Beta diversity of vaginal microbiota across groups. PCoA plots based on (**A**) Bray–Curtis, (**B**) weighted UniFrac, and (**C**) unweighted UniFrac distance matrices illustrating the β-diversity of vaginal microbiota across different study groups. Each colored ellipse represents a 95% confidence interval for the corresponding group. *P*-values calculated using Analysis of Similarities (ANOSIM) are presented in the accompanying tables.

To evaluate beta diversity, PCoA was conducted based on Bray–Curtis, weighted UniFrac, and unweighted UniFrac distance matrices. These complementary metrics were used to capture differences in microbial community structure, composition, and phylogenetic relatedness across groups. Beta-diversity analysis revealed significant differences in microbial community structure between multiple groups. Based on Bray–Curtis distances, the comparisons of NP-VVC vs EP-C (R = 0.118, *P* = 0.016), EP-VVC vs EP-C (R = 0.179, *P* = 0.003), and MP-VVC vs MP-C (R = 0.175, *P* = 0.003) showed statistically significant differences. Weighted UniFrac analysis indicated significant separation between NP-VVC and EP-VVC (R = 0.080, *P* = 0.044), EP-VVC and EP-C (R = 0.113, *P* = 0.014), and MP-VVC and MP-C (R = 0.133, *P* = 0.007). Unweighted UniFrac further demonstrated compositional differences between NP-VVC and EP-C (R = 0.069, *P* = 0.042), MP-VVC and MP-C (R = 0.075, *P* = 0.041), and LP-VVC and LP-C (R = 0.126, *P* = 0.003) (Fig. 3). No significant beta-diversity differences were observed across gestational stages within either healthy controls or VVC-affected women (*P* > 0.05).

### Differentially abundant taxa identified by LEfSe analysis across gestational stages

**Fig 4:**
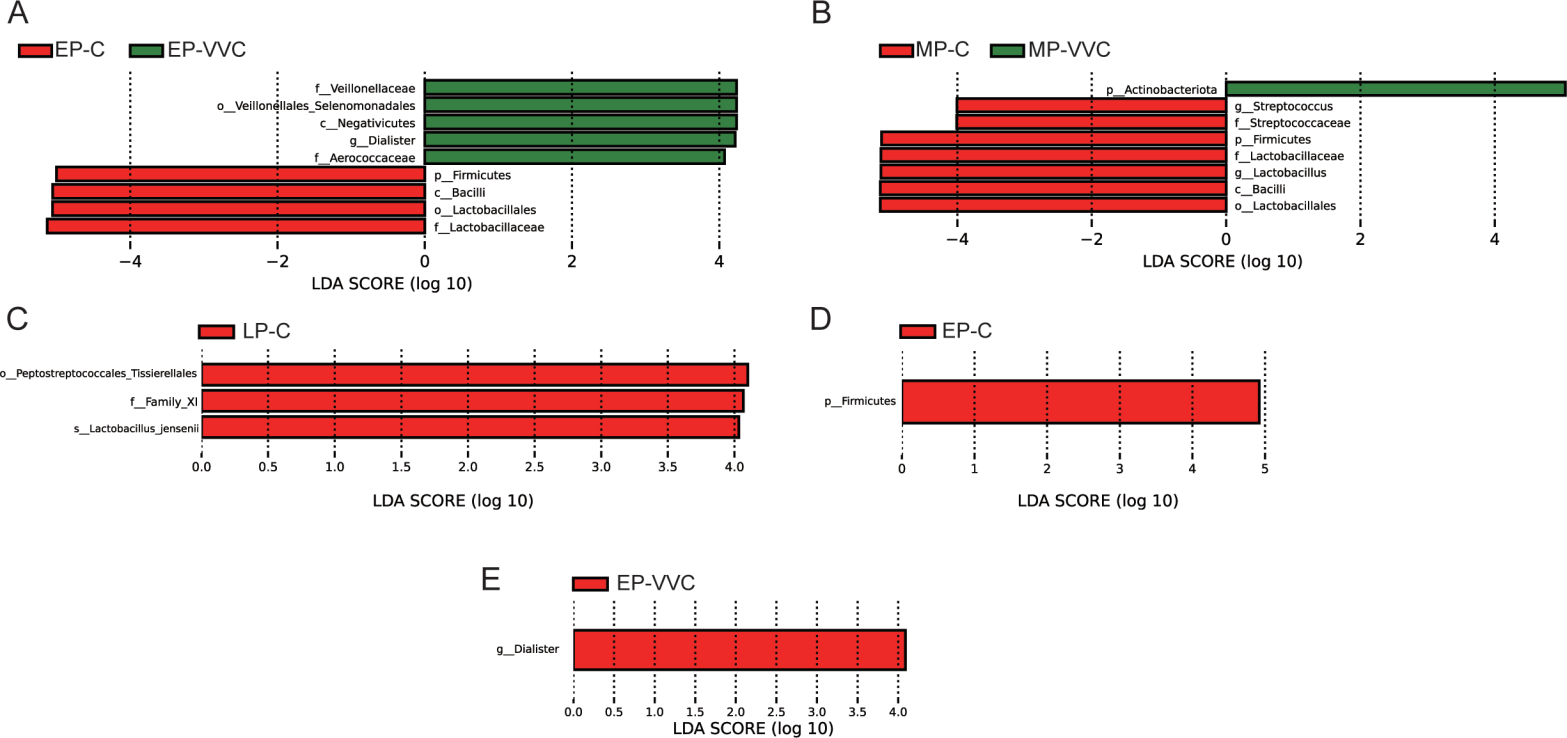
Differential taxa identified by LEfSe analysis across groups. Bar plots showing the different taxa with an LDA score > 4 and *P* < 0.05. The distance between each point represents the degree of difference in the microbiome of each sample. p: Phylum, c: Class, o: Order, f: Family, g: Genus, s: Species. The length of the bars represents the magnitude of the impact of differential species. (**A**) EP-C vs. EP-VVC. (**B**) MP-C vs. MP-VVC. (**C**) LP-C vs LP-VVC. (**D**) EP-C vs MP-C vs LP-C. (**E**) EP-VVC vs MP-VVC vs LP-VVC.

LEfSe analysis was used to identify differentially abundant taxa in the vaginal microbiota between healthy pregnant women and those with VVC at different gestational stages, with the LDA score threshold set at 4 (Fig. 4). In the EP-C group, *Firmicutes* (phylum), *Bacilli* (class), *Lactobacillales* (order), and *Lactobacillaceae* (family) were significantly more abundant than in the EP-VVC group (*P* < 0.05). Conversely, the EP-VVC group showed higher relative abundances of *Veillonellaceae* (family), *Veillonellales_Selenomonadales* (order), *Negativicutes* (class), *Dialister* (genus), and *Aerococcaceae* (family) compared to the EP-C group (*P* < 0.05). In the MP-C group, the taxa *Streptococcus* (genus), *Streptococcaceae* (family), *Firmicutes* (phylum), *Lactobacillaceae* (family), *Lactobacillus* (genus), *Bacilli* (class), and *Lactobacillales* (order) were significantly enriched compared to the MP-VVC group (*P* < 0.05). In contrast, *Actinobacteriota* (phylum) was significantly more abundant in the MP-VVC group than in the MP-C group (*P* < 0.05). Additionally, in the LP-C group, *Peptostreptococcales_Tissierellales* (order), *Family XI* (family), and *Lactobacillus jensenii* (species) were significantly enriched compared to the LP-VVC group (*P* < 0.05). Analysis of healthy pregnant women across gestational stages revealed only an increase in *Firmicutes* (phylum) during early pregnancy (*P* < 0.05). In contrast, among VVC-affected women, *Dialister* (genus) was enriched in mid-pregnancy (*P* < 0.05). Comparison between nonpregnant women with VVC and pregnant women with VVC showed no significant differences in vaginal microbiota composition (*P* > 0.05).

### Network diagram of the correlation of differential microbiota

**Fig 5:**
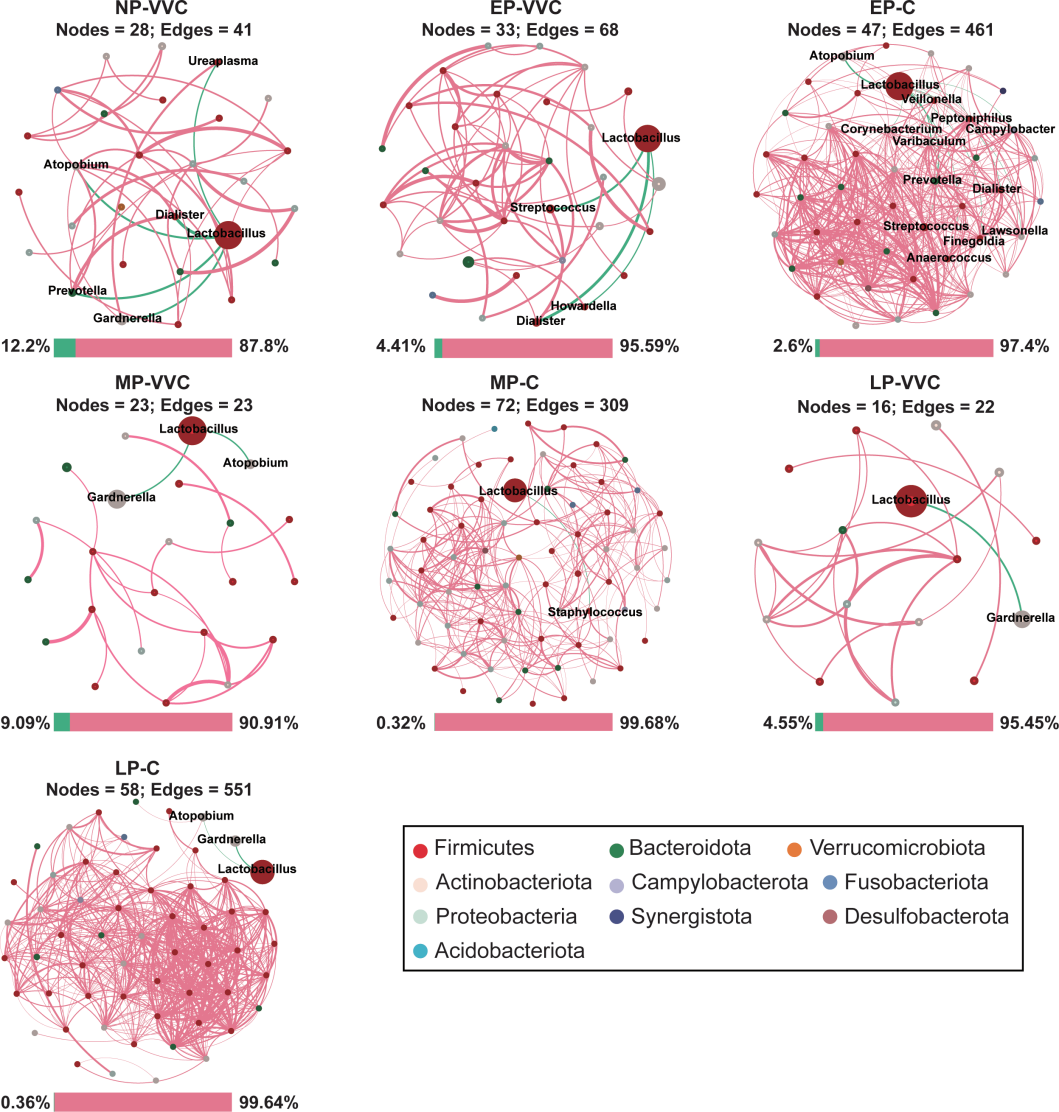
Co-occurrence network analysis for vaginal microbiota. Spearman’s rank correlations were performed, and only associations with |r| ≥ 0.4 and adjusted *P* < 0.05 (Benjamini–Hochberg correction) were retained. The distance between points represents the degree of difference in the microbiota of each sample. Each node represents a genus, the node color represents the phylum, and the node size represents the relative number of OTUs. The connection line represents the presence of a significant correlation between two nodes: a negative correlation indicates a green line, and a positive correlation indicates a red line. The thicker the line is, the greater the Spearman correlation coefficient is between the two nodes. The bar plot below the network shows the proportion of positive and negative correlations.

Network analysis was performed to characterize the structure of the vaginal microbial community across groups. The co-occurrence network in healthy pregnant women was relatively complex, whereas the networks of nonpregnant women with VVC and trimester-specific VVC groups were sparser. Across gestational stages in healthy pregnant women, the interrelationships in the EP-C and LP-C groups were relatively complex, while the MP-C network was relatively simple. In pregnant women with VVC, the EP-VVC network was relatively dense, whereas the MP-VVC and LP-VVC networks were relatively loose (Fig. 5).

**Fig 6:**
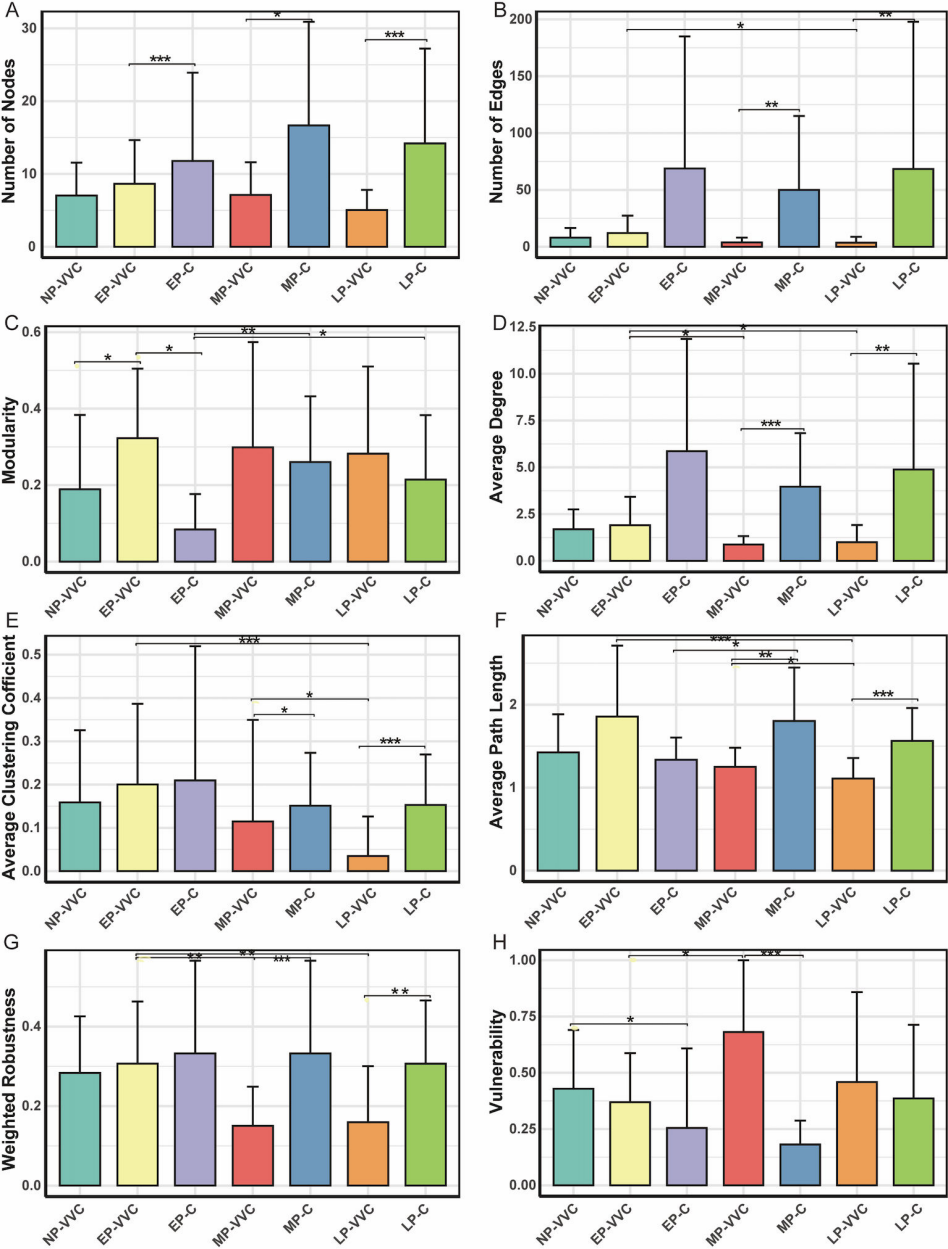
Topological properties of vaginal microbial networks across groups. (**A**) Nodes represent the number of bacterial genera included in each network. (**B**) Edges represent the number of co-occurrence relationships between genera. (**C**) Modularity quantifies the extent to which the network can be divided into distinct modules, with higher values indicating more fragmented communities. (**D**) Average degree reflects the average number of connections per node and indicates overall network complexity. (**E**) The average clustering coefficient measures the degree to which nodes tend to form tightly knit clusters. (**F**) Average path length denotes the average number of steps along the shortest paths for all possible pairs of nodes, reflecting network efficiency. (**G**) Weighted robustness measures the stability of the network under perturbation, with higher values suggesting greater ecological resilience. (**H**) Vulnerability reflects the sensitivity of the network to targeted node removal, with higher values indicating lower robustness and greater susceptibility to disruption. Comparisons between two groups were performed using t-tests or Wilcoxon rank-sum tests, and comparisons among three groups were conducted using one-way ANOVA or Kruskal-Wallis tests. *P < 0.05, **P < 0.01, ***P < 0.001.

Comparisons of network topology revealed significant differences across groups. The number of nodes and edges, average degree, average path length, betweenness centralization, and weighted robustness were higher in MP-C compared to MP-VVC, and in LP-C compared to LP-VVC (*P* < 0.05). Modularity was higher in the EP-VVC network than in EP-C and NP-VVC networks (*P* < 0.05). Vulnerability was increased in MP-VVC compared with MP-C, and in NP-VVC compared with EP-C (*P* < 0.05). Across gestational stages in healthy pregnant women, modularity was higher in MP-C and LP-C than in EP-C (*P* < 0.05). In pregnant women with VVC, EP-VVC exhibited higher numbers of edges, average degree, average path length, betweenness centralization, and weighted robustness than LP-VVC (*P* < 0.05) (Fig. 6).

**Fig 7:**
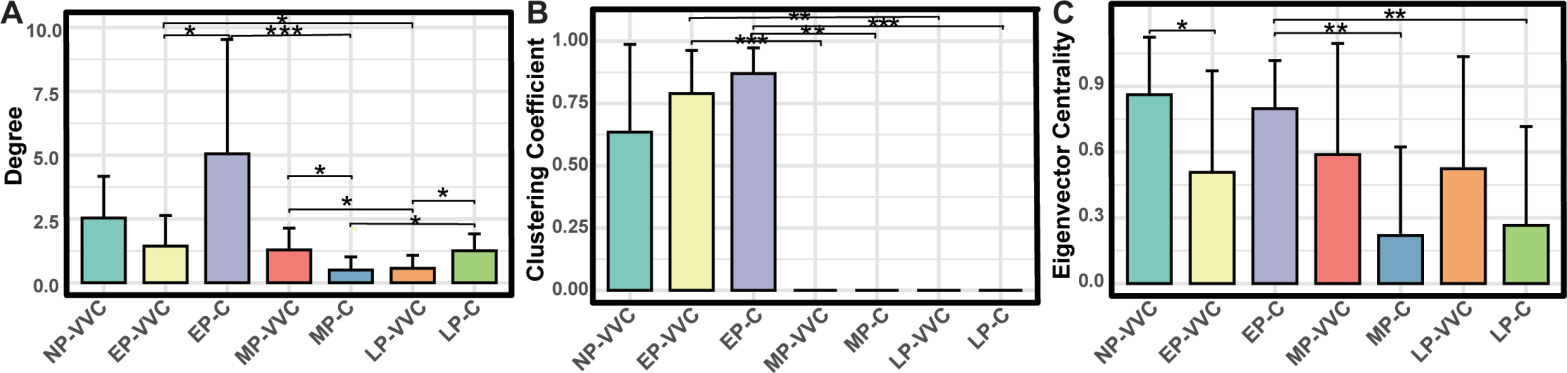
Topological characteristics of *Lactobacillus* in vaginal microbial networks across different groups. (**A**) Degree represents the number of direct connections that *Lactobacillus* has with other genera, indicating its ecological involvement within the microbial community. (**B**) The clustering coefficient measures the extent to which *Lactobacillus* and its neighbors tend to form tightly knit groups, reflecting local cohesiveness. (**C**) Eigenvector centrality evaluates the influence of *Lactobacillus* within the network by considering both the number and quality of its connections; higher values suggest greater centrality and integration within the microbial ecosystem. Comparisons between two groups were performed using t-tests or Wilcoxon rank-sum tests, and comparisons among three groups were conducted using one-way ANOVA or Kruskal-Wallis tests. *P < 0.05, **P < 0.01, ***P < 0.001.

Given that *Lactobacillus* has long been recognized as the most critical genus in maintaining vaginal health, and its supplementation is considered a key strategy for managing VVC during pregnancy, we specifically analyzed the network topological features of *Lactobacillus*. The degree of *Lactobacillus* was higher in the EP-C and LP-C groups than in the EP-VVC and LP-VVC groups, whereas in mid-pregnancy, it was higher in MP-VVC than in MP-C (*P* < 0.05). Across gestational stages in healthy pregnant women, the degree was higher in EP-C and LP-C compared with MP-C, while both the clustering coefficient and eigenvector centrality were higher in EP-C than in MP-C and LP-C (*P* < 0.05). In pregnant women with VVC, both the degree and clustering coefficient were higher in the EP-VVC group than in the LP-VVC group (*P* < 0.05) (Fig. 7). Additionally, *Lactobacillus* exhibited exclusively negative correlations with other genera across all groups (Fig. 5).

### Perinatal outcomes and correlations with abundant vaginal microbiota

Correlation analysis revealed stage-specific associations between vaginal microbiota and perinatal outcomes (Table 3). In EP-C, *Lactobacillus* abundance was positively correlated with fetal weight. In MP-C, *Ligilactobacillus* abundance also showed a positive association with fetal weight, while higher *Lactobacillus* levels were linked to a reduced risk of NICU admission. In contrast, no significant associations between *Lactobacillus* and fetal weight, PROM, or delivery mode were observed in LP-C or in the VVC groups.

**TABLE 3:** Correlations between abundant vaginal microbiota and perinatal outcomes^*a*^

Group	Taxon	Perinatal outcome	r	*P*
EP-VVC	*Atopobium*	Fetal weight	0.51	0.023
	*Veillonella*	Fetal weight	0.46	0.041
	*Fusobacterium*	Fetal weight	0.46	0.041
	*Actinomyces*	NICU admission—yes	0.64	0.002
	*Bacteroides*	NICU admission—yes	0.58	0.008
	*Peptostreptococcus*	1 min Apgar	−0.58	0.008
	*Ureaplasma*	1 min Apgar	−0.45	0.045
	*Staphylococcus*	1 min Apgar	−0.63	0.003
EP-C	*Lactobacillus*	Fetal weight	0.52	0.020
	*Atopobium*	Fetal weight	−0.51	0.020
	*Bacteroides*	Fetal weight	−0.45	0.044
	*Streptococcus*	Amniotic fluid—clear	−0.47	0.034
	*Ezakiella*	Amniotic fluid—clear	−0.47	0.036
MP-VVC	*Bifidobacterium*	Gestational age	0.60	0.006
	*Aerococcus*	Gestational age	0.49	0.032
	*Prevotella_7*	Gestational age	0.54	0.016
	*Dialister*	Delivery mode—cesarean	−0.58	0.009
MP-C	*Howardella*	Gestational age	−0.66	0.002
	*Ezakiella*	Gestational age	−0.51	0.021
	*Porphyromonas*	Gestational age	−0.49	0.027
	*Ligilactobacillus*	Fetal weight	0.49	0.027
	*Lactobacillus*	NICU admission—yes	−0.46	0.044
	*DNF00809*	NICU admission—yes	0.46	0.042
	*Pediococcus*	NICU admission—yes	0.46	0.042
	*Aerococcus*	Delivery mode—cesarean	0.46	0.040
	*Prevotella_7*	Delivery mode—cesarean	0.46	0.040
LP-VVC	*Campylobacter*	Fetal weight	−0.48	0.021
	*Atopobium*	NICU admission—yes	0.60	0.003
	*Dialister*	NICU admission—yes	0.50	0.016
	*Howardella*	NICU admission—yes	0.43	0.040
	*Peptoniphilus*	Amniotic fluid—clear	−0.43	0.042
	*Actinomyces*	PROM—yes	0.46	0.025
	*Ezakiella*	PROM—yes	0.46	0.025
	*Staphylococcus*	PROM—yes	0.46	0.025
	*Megamonas*	PROM—Yes	0.46	0.025
	*Stenotrophomonas*	Delivery mode—cesarean	0.50	0.014
LP-C	*Prevotella*	Gestational age	−0.49	0.027
	*Gardnerella*	Fetal weight	0.45	0.048
	*Aerococcus*	Fetal weight	0.45	0.046
	*Dialister*	Amniotic fluid—clear	0.46	0.040
	*Atopobium*	Delivery mode—cesarean	0.55	0.013
	*Howardella*	Delivery mode—cesarean	0.54	0.013
	*Anaerococcus*	Delivery mode—cesarean	0.61	0.004
	*Ezakiella*	Delivery mode—cesarean	0.59	0.007
	*Limosilactobacillus*	Delivery mode—cesarean	0.57	0.009
	*Porphyromonas*	Delivery mode—cesarean	0.59	0.007
	*Peptoniphilus*	Delivery mode—cesarean	0.75	<0.001
	*Peptostreptococcus*	Delivery mode—cesarean	0.57	0.009
	*Campylobacter*	Delivery mode—cesarean	0.65	0.002
	*Finegoldia*	Delivery mode—cesarean	0.64	0.002
	*Murdochiella*	Delivery mode—cesarean	0.57	0.009
	*Parvimonas*	Delivery mode—cesarean	0.45	0.045
	*Staphylococcus*	Delivery mode—cesarean	0.54	0.013
	*Sutterella*	Delivery mode—cesarean	0.46	0.044
	*Anaeroglobus*	Delivery mode—cesarean	0.45	0.045
	*S5-A14a*	Delivery mode—cesarean	0.45	0.045
	*Negativicoccus*	Delivery mode—cesarean	0.45	0.045
	*Blautia*	Delivery mode—cesarean	0.45	0.045
	*Fusicatenibacter*	Delivery mode—cesarean	0.45	0.045

^*a*^ Spearman’s rank correlation analysis was conducted between perinatal outcomes and the top 50 genera in terms of relative abundance within each group. Only correlations with |r| ≥ 0.4 and an adjusted *P* < 0.05 (Benjamini–Hochberg correction) were considered significant.

## DISCUSSION

This study provides a comprehensive characterization of the vaginal microbiota in women with VVC across nonpregnant and pregnant states, including different gestational stages. By integrating taxonomic composition, diversity metrics, differential abundance analysis, and microbial ecological network modeling, we identified distinct microbial patterns associated with both pregnancy and VVC status. Notably, we observed substantial differences in microbial diversity and structure between VVC and non-VVC groups, as well as across different trimesters. In addition, we found that *Lactobacillus*, a keystone genus for vaginal health, exhibited dynamic changes in both abundance and ecological connectivity during pregnancy, suggesting a possible link between gestational progression and microbial resilience. These findings offer new insights into the interaction between host physiological states and vaginal microbiota dysbiosis in the context of VVC.

Our taxonomic analysis revealed that *Firmicutes*, *Actinobacteria*, and *Bacteroidetes* were the predominant phyla in most groups, with *Firmicutes* consistently dominant. However, both *Actinobacteria* and *Bacteroidetes* displayed notable fluctuations between pregnant and nonpregnant states and across trimesters. At the genus level, *Lactobacillus* remained the most abundant taxon, though its relative abundance declined during mid to late pregnancy, particularly in VVC patients. Species-level profiling within the genus *Lactobacillus* revealed distinct temporal patterns: *Lactobacillus_iners* increased slightly in mid-pregnancy but decreased in late pregnancy, while remaining more abundant in VVC patients than in gestational age-matched controls; in contrast, *Lactobacillus_jensenii* decreased in mid-pregnancy but increased toward late pregnancy. These patterns likely reflect functional differences among *Lactobacillus* species: *L. iners* is considered a transitional species, producing only L-lactic acid and inerolysin, with a limited metabolic repertoire and the ability to coexist with bacteria associated with bacterial vaginosis, rendering it less protective (28). By contrast, *L. jensenii* produces both D- and L-lactic acid and bacteriocins, which can inhibit *Candida* growth or hyphal transition (9). Additionally, the abundance of *Gardnerella* increased with advancing gestation, paralleled by an increase in VVC cases among gestational age-matched patients, suggesting a shift toward dysbiosis. These microbial changes may reflect hormone-driven alterations in the vaginal microenvironment. Estrogen levels rise sharply during the first trimester and remain stable throughout the second and third trimesters. This sustained high-estrogen environment promotes proliferation of the vaginal epithelium and glycogen deposition, which is metabolized by *Lactobacillus* species into lactic acid, maintaining a protective low pH and shaping microbial composition to suppress opportunistic organisms (29, 30). Beyond hormonal regulation, the vaginal microbiota is also shaped by host immune status and ecological interactions within the community (31). The interplay of these factors may account for the dynamic microbial shifts observed across pregnancy stages.

Chao1 and observed species indices primarily reflect species richness, whereas Shannon and Simpson indices account for both richness and evenness. Alpha diversity analysis revealed distinct temporal patterns across gestational stages. In early and mid-pregnancy, VVC groups exhibited significantly higher Shannon and Simpson indices than healthy controls, indicating increased diversity in terms of community evenness. This increase may be attributed to the disruption of *Lactobacillus*-dominated communities during infection, allowing multiple anaerobic or opportunistic taxa to expand and achieve a more balanced distribution. This observation is consistent with previous findings (32). However, it is important to note that the vaginal microbiota undergoes continuous shifts throughout pregnancy (12). In late pregnancy, the LP-C group showed higher Chao1 and Observed species indices than the LP-VVC group, indicating greater microbial richness in healthy individuals. This may reflect the establishment of a more mature and diverse microbial community in the absence of infection. In contrast, VVC during late pregnancy may interrupt this natural progression, limiting increases in microbial richness. Similarly, while healthy women showed a significant increase in Shannon and Simpson indices from mid- to late pregnancy, indicating enhanced community evenness, this maturation of microbial balance was notably absent in VVC groups. Collectively, these results suggest that VVC exerts gestational stage-dependent effects on the vaginal microbiota.

Beta-diversity analysis further confirmed distinct microbial structures across groups. Significant group separation was observed in Bray–Curtis, weighted UniFrac, and unweighted UniFrac analyses, particularly between matched VVC and non-VVC groups within the same trimester, whereas overall beta diversity did not differ significantly across gestational stages. These findings suggest that VVC is associated with altered microbial composition and phylogenetic structure, potentially providing microbiome-based biomarkers for diagnosis.

LEfSe analysis across different gestational stages consistently highlighted the decrease in *Lactobacillus* and its related taxa—such as *Lactobacillaceae*, *Lactobacillales*, and *Bacilli*—as a hallmark of VVC-associated vaginal microbiota. In all three trimesters, healthy pregnant women exhibited significant enrichment of *Lactobacillus*, underscoring its central role in maintaining vaginal homeostasis during pregnancy. This pattern reinforces the notion that *Lactobacillus* dominance is a defining feature of a healthy vaginal ecosystem and that its disruption is a common microbial signature of VVC (33, 34), regardless of gestational age. Collectively, these findings underscore that the reduction of *Lactobacillus* may serve as a key microbial marker for identifying VVC during pregnancy. Our study also revealed distinct temporal dynamics in vaginal microbial composition. In healthy pregnancies, early gestation is characterized by an initial enrichment of *Firmicutes*, likely reflecting the early establishment of a *Lactobacillus*-dominated microbiota that supports mucosal protection and microbial stability. Interestingly, no significant compositional differences were observed between nonpregnant and pregnant women with VVC, suggesting that the development of VVC may override physiological differences associated with pregnancy, driving the microbiota toward a convergent dysbiotic configuration.

Network-level analysis revealed notable shifts in the structural properties of the vaginal microbial community across different gestational stages and health statuses. Microbial co-occurrence networks reflect underlying ecological interactions; decreased connectivity and increased modularity may indicate fragmentation of the microbial ecosystem, potentially reducing colonization resistance against pathogens (35). In healthy pregnant women, particularly during mid- to late pregnancy, the vaginal microbiota exhibited higher numbers of nodes and edges, greater average degree, and increased robustness, suggesting a more interconnected and stable microbial network. In contrast, women with VVC displayed lower values for these parameters, indicating a simplified and potentially destabilized network structure. Given that VVC has been implicated in preterm birth (36) and neonatal infections (37), the observed microbial instability in mid- and late-pregnancy VVC cases may have broader implications for maternal-fetal outcomes. Stage-specific alterations were also evident. In early pregnancy, modularity was higher in EP-VVC than in EP-C, suggesting a more fragmented but ecologically compartmentalized network, whereas in mid-pregnancy, vulnerability was higher in MP-VVC compared with healthy women, reflecting reduced resilience to perturbations. These findings suggest that VVC is associated with a breakdown in microbial connectivity, leading to communities that are less cohesive and potentially more susceptible to perturbations or pathogen overgrowth. Importantly, network differences between healthy and VVC groups were present at all gestational stages, although the specific divergent metrics varied, highlighting the systemic ecological impact of VVC on the vaginal microbiota. Considering the entire gestational trajectory, healthy pregnant women showed a significant increase in modularity during mid-pregnancy, which remained relatively stable in late pregnancy. In contrast, this pattern was absent in women with VVC. Their networks were more complex in early pregnancy, suggesting that VVC disrupts the normal organizational trajectory of the microbiota and leads to reduced connectivity and stability in later stages of pregnancy.

*Lactobacillus* species are widely recognized as key members of the vaginal microbiota, contributing to the maintenance of homeostasis through lactic acid production for pH regulation, inhibition of pathogenic microorganisms, and stabilization of community composition via extracellular vesicles (38–40). Emerging evidence indicates that pregnancy-related immune tolerance interacts closely with the vaginal microbiota (41). Conversely, *Lactobacillus* depletion has been linked to higher microbial diversity, reduced antimicrobial peptides (e.g., β-defensin-2) (42), and enhanced inflammatory responses, which may increase risks such as preterm labor. Given the pivotal role of *Lactobacillus* in vaginal health, we examined its topological features within the microbial network. The results showed that *Lactobacillus* occupied a more central position in healthy networks, with a higher degree observed in early and late pregnancy compared with corresponding VVC groups. In mid-pregnancy, the degree was higher in MP-VVC than in MP-C, indicating that the network role of *Lactobacillus* varies with both gestational stage and health status. Additionally, temporal patterns such as higher degree, clustering coefficient, and eigenvector centrality in early pregnancy suggest that the vaginal microbial network undergoes dynamic structural changes throughout gestation. These findings highlight the importance of considering both gestational stage and infection status when evaluating *Lactobacillus* within the vaginal microbial community.

Correlation analysis revealed stage-specific associations between vaginal microbiota and perinatal outcomes in healthy pregnant women. In early and mid-pregnancy, the abundance of *Lactobacillus* or *Ligilactobacillus* was positively associated with fetal weight, suggesting that a healthy maternal vaginal microenvironment may support optimal fetal growth. Additionally, higher *Lactobacillus* levels in mid-pregnancy were linked to a reduced risk of NICU admission, further highlighting its protective role. These findings are consistent with previous studies reporting that *Lactobacillus*-dominated vaginal communities are associated with improved birth outcomes (43), emphasizing the importance of maternal vaginal microbiota composition in perinatal health.

Several limitations should be acknowledged in this study. First, this was a cross-sectional study rather than a longitudinal one, and participants were grouped by gestational stage rather than being followed throughout pregnancy. As such, individual microbial trajectories and intra-individual dynamics could not be assessed. Second, this study did not include a completely healthy nonpregnant control group, as all nonpregnant participants were recruited with VVC. While this design allowed comparison under similar symptomatic conditions, it limits direct baseline comparison with healthy nonpregnant women, which should be addressed in future studies. Third, VVC diagnosis was based on wet mount microscopy, which has limited sensitivity, particularly for low-level infections or non-hyphal *Candida* forms. Fourth, we did not directly measure serum hormone levels, dietary patterns, or sexual activity frequency, all of which may influence changes in the vaginal microbiota. Fifth, although we included samples from both pregnant and nonpregnant women with VVC, the number of participants within each subgroup was relatively limited, which may reduce statistical power and generalizability. Sixth, while 16S rRNA gene sequencing allowed for profiling of bacterial communities, it does not capture fungal species or the functional potential of the microbiota. Given the key role of *Candida* in VVC pathogenesis, future multi-omics studies, including ITS sequencing, shotgun metagenomics, and metabolomics, will be required to validate these findings at higher taxonomic resolution and to comprehensively evaluate host–microbe–fungus interactions (44).

### Conclusion

In summary, this study provides a comprehensive analysis of the vaginal microbiota in VVC-affected women across nonpregnant and different gestational stages, highlighting both compositional and ecological differences shaped by physiological states. We found that the impact of VVC on the vaginal microbiome varies with gestational age, as infections at different stages exhibited distinct diversity patterns. Network analysis further revealed that VVC disrupts microbial connectivity and stability, particularly in mid- and late pregnancy, and reduces the connectivity of *Lactobacillus*, especially in early and late pregnancy (Table 4). Vaginal microbial networks undergo dynamic structural changes across gestation, which can be perturbed by VVC. These findings suggest that VVC is not a uniform condition but rather a dynamic dysbiosis influenced by host physiology, and that treatment strategies may need to be tailored by gestational stage. Future longitudinal and multi-omics studies are warranted to validate these insights and inform precision approaches for VVC prevention and management in pregnancy.

**TABLE 4:** Dynamic changes in vaginal microbiota characteristics between VVC patients and healthy controls across gestational stages^*a*^

Characteristic	Group	Early pregnancy	Mid-Pregnancy	Late pregnancy
Alpha diversity	HC	→	→	↑ (Chao1, Observed_species)
	VVC	↑ (Shannon, Simpson)	↑ (Shannon, Simpson)	→
Beta diversity	VVC vs. HC	Significantly different	Significantly different	Significantly different
Key Taxa (LEfSe)	HC	Enriched: *Lactobacillales*, *Lactobacillaceae*	Enriched: *Lactobacillus*, *Streptococcus*	Enriched: *L. jensenii*
	VVC	Enriched: *Dialister*, *Aerococcaceae*	Enriched: *Actinobacteriota*	/
Network topology	HC	→ Complexity	↑ Complexity	↑ Complexity
	VVC	↑ Modularity, → Complexity	↓ Complexity	↓ Complexity
*Lactobacillus* connectivity	HC	High (degree)	Low	High (degree)
	VVC	Low	High (degree)	Low

^*a*^ ↑, Increased compared to the corresponding group within the same trimester; ↓, decreased; →, no significant change. HC, healthy controls.

## Data Availability

All data generated or analyzed during this study are included in this published article. The sequence data reported in this study were archived in the Sequence Read Archive with the accession number PRJNA1307019.

## SUPPLEMENTAL MATERIAL

Supplemental tables spectrum.02144-25-s0001.xlsx Tables S1 to S4.

ASM does not own the copyrights to Supplemental Material that may be linked to, or accessed through, an article. The authors have granted ASM a non-exclusive, world-wide license to publish the Supplemental Material files. Please contact the corresponding author directly for reuse.
